# cAMP-dependent and cholinergic regulation of the electrogenic intestinal/pancreatic Na^+^/HCO_3_^- ^cotransporter pNBC1 in human embryonic kidney (HEK293) cells

**DOI:** 10.1186/1471-2121-9-70

**Published:** 2008-12-22

**Authors:** Oliver Bachmann, Kristin Franke, Haoyang Yu, Brigitte Riederer, Hong C Li, Manoocher Soleimani, Michael P Manns, Ursula Seidler

**Affiliations:** 1 Dept. of Gastroenterology, Hepatology, and Endocrinology, Hannover Medical School, Hannover, Germany; 2 Division of Nephrology and Hypertension, Dept. of Internal Medicine, University of Cincinnati, OH, USA; 3 Institute of Cell Biology and Neurobiology, Charité, Berlin, Germany

## Abstract

**Background:**

The renal (kNBC1) and intestinal (pNBC1) electrogenic Na^+^/HCO_3_^- ^cotransporter variants differ in their primary structure, transport direction, and response to secretagogues. Previous studies have suggested that regulatory differences between the two subtypes can be partially explained by unique consensus phosphorylation sites included in the pNBC1, but not the kNBC1 sequence. After having shown activation of NBC by carbachol and forskolin in murine colon, we now investigated these pathways in HEK293 cells transiently expressing a GFP-tagged pNBC1 construct.

**Results:**

Na^+^- and HCO_3_^-^-dependent pH_i _recovery from an acid load (measured with BCECF) was enhanced by 5-fold in GFP-positive cells compared to the control cells in the presence of CO_2_/HCO_3_^-^. Forskolin (10^-5 ^M) had no effect in untransfected cells, but inhibited the pH_i _recovery in cells expressing pNBC1 by 62%. After preincubation with carbachol (10^-4 ^M), the pH_i _recovery was enhanced to the same degree both in transfected and untransfected cells, indicating activation of endogenous alkalizing ion transporters. Acid-activated Na^+^/HCO_3_^- ^cotransport via pNBC1 expressed in renal cells is thus inhibited by cAMP and not affected by cholinergic stimulation, as opposed to the findings in native intestinal tissue.

**Conclusion:**

Regulation of pNBC1 by secretagogues appears to be not solely dependent on its primary structure, but also on properties of the cell type in which it is expressed.

## Background

The electrogenic Na^+^/HCO_3_^-^-cotransporter isoform 1 (NBC1) is basolaterally expressed in the renal proximal tubule, where it mediates HCO_3_^- ^reabsorption by concerted action with the apical Na^+^/H^+^-exchanger isoform NHE3 [1], and in gastrointestinal epithelia, where it serves the intracellular supply of HCO_3_^- ^destined for secretion [2]. These striking differences in function and transport direction have prompted studies to elucidate the structural and regulatory properties of the respective transporters. It was found that renal NBC is inhibited by an increase in intracellular cAMP [1], enabling the parallel regulation with NHE3, which is also inhibited in a cAMP-dependent manner [3]. In contrast, we could previously show that forskolin stimulates intestinal NBC [4]. However, exposure to cholinergic compounds causes an increase of both the renal and the intestinal Na^+^/HCO_3_^- ^cotransporter rates [5,6].

One explanation for the differential regulation of Na^+^/HCO_3_^- ^cotransport in these tissues comes from the identification of structurally distinct splice variants of NBC1. The renal (kNBC1) and intestinal (pNBC1) NBC subtypes possess a common C-terminal PKA-dependent phosphorylation site (Ser^982 ^and Ser^1026^, respectively), which was reported to determine transport stoichiometry in renal cells [7,8]. In addition, however, the longer N-terminal tail of pNBC1 contains unique phosphorylation sites for PKA (Thr49), PKC (Ser^38 ^and Ser^65^), and casein kinase II (Ser^68^), which are not found in the kNBC1 sequence and of which at least the cAMP-dependent site is relevant for transporter regulation [7,9].

On the other hand, there is increasing evidence that the cell type plays a central role in determining how ion transport is regulated [10-13]. As knowledge of intestinal Na^+^/HCO_3_^- ^cotransporter function and regulation is overall limited, this important aspect has not been studied in great detail. There is only one report on cAMP-dependent stoichiometry changes of heterologously transfected pNBC1 involving the common C-terminal phosphorylation site [7]. However, information on cell-type dependency of intestinal NBC regulation by secretagogues during its presumed physiological function, which is HCO_3_^- ^uptake in the process of anion secretion [2], is lacking. We therefore set off to investigate HCO_3_^- ^import via pNBC1 transfected into HEK293 cells in acidification experiments. The aim of the study was to clarify whether regulation of heterologously transfected pNBC1 by secretagogues is similar as in native colonic tissue and thus essentially dependent on structural determinants of the transporter protein, or different and thus affected by the cell type in which it is expressed.

## Results

**Figure 1:**
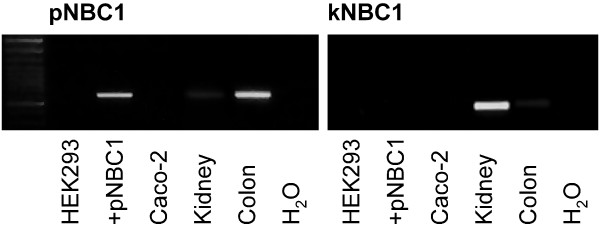
**RT-PCR in untransfected HEK293 cells (HEK293), HEK293 cells transiently transfected with pNBC1 (+pNBC1), as well as human kidney and colon samples using kNBC1- and pNBC1-specific primers (see methods section)**. While neither isoform was detected in untransfected HEK293 cells, a pNBC1 fragment of the expected size (612 bp) was amplified from transfected HEK293 cells and human colon. kNBC1 was exclusively detected in human kidney samples (expected PCR product size: 489 bp). H_2_O indicates the reaction where water was used as a template.

To determine the distribution of NBC1 subtypes in HEK293 cells compared to native tissue, we first performed PCR analysis (Figure 1). Neither pNBC1 nor kNBC1 mRNA was amplified from untransfected HEK293 cells. As anticipated, pNBC1-specific primers detected a signal in HEK293 cells transiently transfected with pNBC1, and in human colon. kNBC1 was detected in human kidney and, to a lesser degree, in human colon samples.

**Figure 2:**
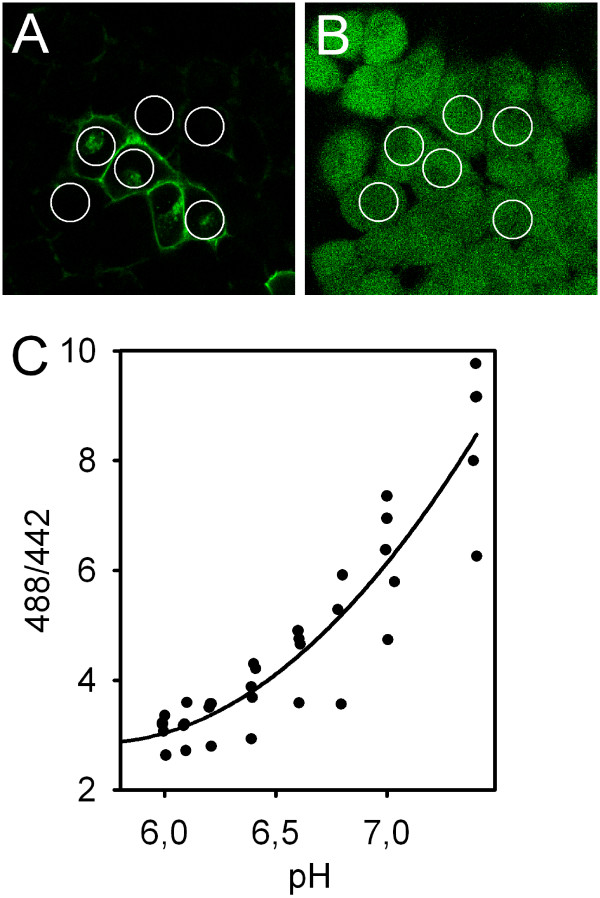
**Confocal images of HEK cells transiently transfected with GFP-pNBC1**. Panel (A) shows the GFP staining of transfected cells and the weak autofluorescence of untransfected cells. After placing regions of interest covering most of the cytosol, cells were in situ loaded with the pH-sensitive dye BCECF (B), and the time course of the intracellular pH was recorded. C: To determine the optimal calibration procedure for the pH range of interest (for calculation of the recovery rates at the reference pH_i_, see Fig. 3B), a calibration curve was constructed using high K^+^/nigericin solution to clamp intracellular pH at different levels. As expected, its slope is less steep at low pH_i _values and steeper in the linear range (n = 6). To minimize the error during the calibration procedure within the pH range of interest, the calibration points 6.2 and 7.0 were chosen. Since the ratio values varied considerably between the experiments, each individual region was calibrated after the respective experiment subsequently.

**Figure 3:**
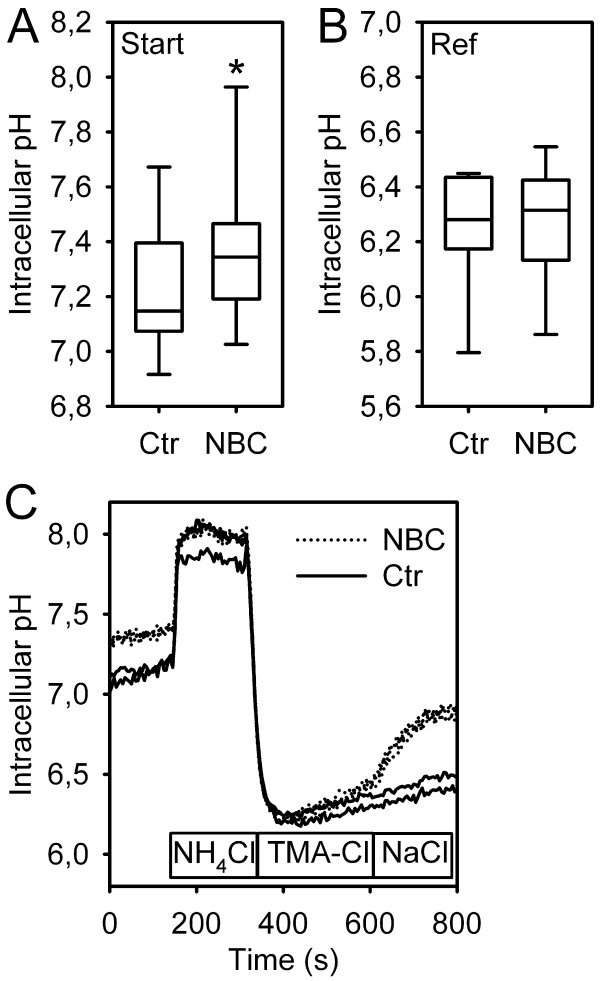
**Starting pH_i_, reference pH_i_, and representative pH_i _tracings from pNBC1-transfected and untransfected HEK293 cells**. A, B: Box and whisker diagram of the starting- and reference pH_i _values from pNBC1-transfected and untransfected cells. Steady-state pH_i _was significantly higher in transfected cells, pointing to increased baseline Na^+^/HCO_3_^- ^cotransport activity (*: p < 0.001, Student's t-test for paired samples, n = 7). The reference pH_i _after acidification was, however, not different between the two. C: After imposing an acid load using the "NH_4_-prepulse" method, cells acidified to a similar extent. Upon Na^+ ^readdition, most untransfected cells showed no significant recovery, while pH_i _increased rapidly in transfected cells (representative tracings). It should be noted that our 2-point calibration approach is optimized for the low pH_i _values at which transporter activity is measured, and that the higher pH_i _values are therefore overestimated.

Next, transiently transfected HEK293 cells were visualized using confocal microscopy (Figure 2A). The transfection efficiency was consistently at 10–15% of the cells. After selecting regions of interest (ROIs) covering the major part of the cytoplasm, the image was digitized for documentation, and cells were *in situ *loaded with BCECF (Figure 2B). Two groups of cells were identified: Cells with a strong GFP signal along the cell membrane (subsequently called "GFP-positive" or "transfected"), and cells with no or very faint fluorescence in this location (subsequently called "GFP-negative" or "untransfected"). To exclude cells with intermediate GFP signal which only weakly express the transfected construct, the signal was quantified by placing a rectangular plot profile with vertical averaging over at least 20 pixels (for noise reduction) perpendicularly over the cell membrane using ImageJ (NIH, Bethesda, MD, USA, ) under standardized illumination settings [14]. Indeed, the first group of cells exhibited fluorescence intensity values of 98.8 ± 14.2 AU (arbitrary units), and the second group values of 17.0 ± 3.1 AU (p < 0.01 vs. first group, not significantly different from the background); the amount of cells with intermediate GFP signal was low (<10%), and these cells were not used for the functional experiments. Cells were then subjected to an NH_4_^+^-prepulse protocol to impose an acid load in the presence of CO_2_/HCO_3_^- ^(Figure 3C). It is important to note in this context that the high pH_i _values achieved during the prepulse are overestimated, since our calibration approach is optimized for the low pH_i _values where transporter activity is actually measured. pH_i _recovery upon readdition of Na^+ ^occurred faster in GFP-positive than in GFP-negative cells. Since the steady-state pH_i _appeared to be higher in transfected cells, we statistically compared the obtained values from untransfected and transfected cells, which indeed demonstrated a significantly more alkaline pH_i _in pNBC1-transfected cells, pointing to increased basal NBC activity (Figure 3A). The reference pH_i_, however, was not significantly different between the two (Figure 3B). Overall, the correlation between GFP signal intensity and recovery rates were excellent, demonstrating the correct identification of the transfected cells. The resting pH_i _and the reference pH_i _of mock-transfected cells were equal as in untransfected cells (not shown).

**Figure 4:**
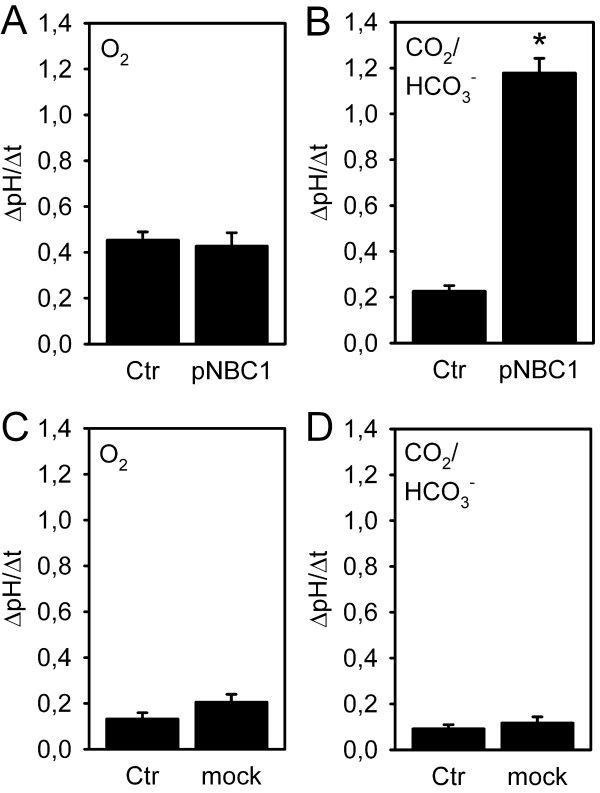
**pH_i _recovery from an acid load in untransfected (Ctr) and transfected cells (pNBC1, mock) in the absence and presence of CO_2_/HCO_3_^-^**. A: in O_2_/HEPES buffer, no significant difference in the pH_i _recovery rate in untransfected and pNBC1-transfected cells was noted (n = 8). B: In the presence of CO_2_/HCO_3_^-^, however, pH_i _was accelerated 5-fold in transfected vs. untransfected HEK293 cells (*: p < 0.05, Student's t-test for paired samples, n = 7), indicating successful functional transfection of the pNBC1 protein. C, D: in mock-transfected vs. untransfected cells on the same slide, pH_i _recovery rates were relatively low, and no significant differences were noted between mock-transfected and untransfected, or between the absence and presence of CO_2_/HCO_3_^-^, respectively (n = 6–8).

To determine whether pNBC1 transfected in HEK293 cells is functional, pH_i _recovery rates were quantified in untransfected and transfected cells in the presence and absence of CO_2_/HCO_3_^- ^(Figure 4). In HEPES buffered solution, no significant difference in ΔpH/Δt was observed, indicating no Na^+^/HCO_3_^-^-cotransport in HEK293 cells due to the lack of substrate (A). The alkalization under these conditions is most likely mediated by the Na^+^/H^+^-exchanger, of which the NHE1 and NHE3 isoforms are expressed in HEK293 cells [15,16]. In the presence of CO_2_/HCO_3_^-^, however, pH_i _recovery occurred significantly faster at a 5-fold higher rate in transfected vs. untransfected cells (B). This finding clearly demonstrates successful functional transfection of pNBC1. ΔpH/Δt appeared somewhat lower in untransfected cells in the presence than in the absence of CO_2_/HCO_3_^-^, probably due to a higher buffering capacity in its presence, but this difference did not reach statistical significance. To exclude that the GFP signal significantly influences the recovery rates, control experiments using mock-transfected HEK cells were carried out (C, D). Here, recovery rates were generally low, and not significantly different between mock-transfected vs. untransfected in the presence and absence of CO_2_/HCO_3_^-^, respectively.

**Figure 5:**
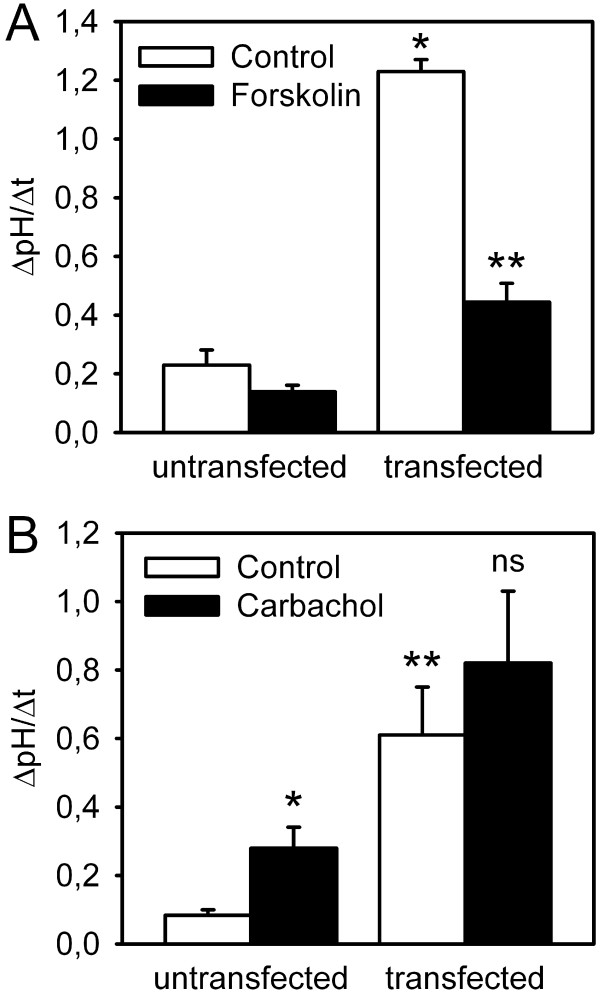
**Effect of cAMP-dependent and cholinergic stimulation on pH_i _recovery in untransfected and pNBC1-transfected HEK293 cells**. A: Forskolin did not elicit any significant changes in pH_i _recovery in untransfected cells (n = 6). Again, transfected cells displayed a significantly faster pH_i _recovery under control conditions than their untransfected counterparts (*: p < 0.05 vs. untransfected control, Student's t-test for paired samples, n = 6). However, forskolin strongly inhibited pH_i _recovery in pNBC1-transfected HEK293 cells (**: p < 0.01 vs. unstimulated control, Student's t-test for unpaired samples, n = 7). B: Carbachol caused a slight acceleration of pH_i _recovery in untransfected cells, which might be due to effects on endogenous Na^+^/H^+ ^exchange or on Na^+^/HCO_3_^- ^exchanger isoforms other than pNBC1 or kNBC1 (*: p < 0.05 vs. unstimulated control, Student's t-test for unpaired samples, n = 6). pNBC1 transfection again led to a significantly faster pH_i _recovery (**: p < 0.05 vs. untransfected control, Student's t-test for paired samples, n = 8). However, carbachol did not cause any additional significant changes to pH_i _recovery in transfected cells (ns: not significant, n = 8).

In our previous work, we had delineated the effects of secretagogues on endogenous Na^+^/HCO_3_^-^-cotransport, which is most likely effectuated predominantly by the pNBC1 subtype of the electrogenic isoform NBC1, in murine proximal colonic crypts. In accordance with the concept of NBC as an anion importer during cAMP-dependent and cholinergic stimulation of anion secretion, we could demonstrate transporter activation by both types of secretagogues [4,6]. We now set off to investigate the influence of the cell type on cotransporter regulation by secretagogues, and therefore studied the effect of forskolin and carbachol on pNBC1 transfected into HEK293 cells as a non-intestinal cellular model. As depicted in figure 5A, an increase in intracellular cAMP did not significantly change pH_i _recovery rates in untransfected cells, indicating that this measure possibly causes differential, and balanced effects on the Na^+^/H^+ ^exchanger isoforms expressed in HEK293 cells [15,16] and/or on endogenous NBC. However, forskolin stimulation significantly decreased pH_i _recovery rate in pNBC1-transfected HEK293 cells by more than 50%, which is in sharp contrast to the findings in native tissue [4,7].

HEK293 cells have been shown functionally express the M3 subtype of cholinergic receptors [17], which mediates NBC activation by carbachol in murine colonic crypts [6]. When untransfected cells were subjected to cholinergic stimulation, ΔpH/Δt was increased compared to the control, which points to the activation of NHE1 which is expressed in HEK293 cells. In pNBC1-transfected cells, however, carbachol had no additional stimulatory impact on pH_i _recovery and did not cause changes in pH_i _recovery altogether (Figure 5B). Given its effect on untransfected cells, carbachol does not stimulate NBC, but might even have an inhibitory effect on this transport system. This finding represents another discrepancy of endogenous intestinal versus heterologously transfected pNBC1 regulation [6].

## Discussion

The renal and intestinal Na^+^/HCO_3_^- ^cotransporters are differentially regulated by cAMP-dependent and cholinergic agonists [1,4-6]. Possible explanations for these findings are differences in their primary structure influencing regulatory properties, or features of the cell type in which they are expressed. In this study, we investigated the secretagogue regulation of the intestinal-pancreatic NBC subtype pNBC1 in HEK293 cells.

The group of Ira Kurtz has thoroughly studied the stoichiometry of NBC1 and found that the renal subtype kNBC1 is transporting 1 Na^+ ^and 3 HCO_3_^- ^with each cycle, which results in outward-directed transport [8]. Functional inhibition of kNBC1 by cAMP is probably in part due to a stoichiometry shift from 3:1 to 2:1, leading to a switch from export to import. On the other hand, the intestinal subtype pNBC1 serves the uptake of both ions with a 2:1 ratio under physiological conditions [18], and a stoichiometry change was not reported for the endogenously expressed transporter. However, pNBC1 can be activated via cAMP [4], which is due to the phosphorylation of a unique consensus phosphorylation site in its N-terminus [7]. As to a structure-function relationship regarding cholinergic stimulation, which increases the transport rate of both subtypes in a PKC-dependent manner [5,6], the possible relevance of the unique PKC-dependent phosphorylation site in the pNBC1 N-terminus remains to be assessed.

Importantly, cell type- and tissue-specific regulation has been recognized for many ion transporters including Na^+^/H^+^-exchange [10,11], anion exchange [12], and CFTR [13]. Expression of pNBC1 and kNBC1 is not restricted to the pancreas/intestinal tract and the kidney, respectively, and expression in other organs such as the eye [19], the gallbladder [20], and salivary glands [21] has been reported, but it has not been investigated whether these different cell types modulate transporter regulation. Gross et al. have previously studied the regulation of murine pNBC1 endogenously occurring in pancreatic duct cells and heterologously transfected into a mouse proximal tubule cell line [7]. They found that cAMP causes upregulation of endogenous pNBC1 via one PKA-dependent phosphorylation site not present in the kNBC1 sequence, while changing the stoichiometry from 3:1 to 2:1 and thereby transport direction via another common C-terminal site (Ser^982 ^and Ser^1026 ^for kNBC1 and pNBC1, respectively), the latter resembling kNBC1 regulation. In our experiments, however, we sought to clarify the cell-type dependency of the regulation of the presumed physiological function of pNBC1, which is HCO_3_^- ^uptake during stimulated anion secretion, and which can be activated by imposing an acid load on the cytosol. In this setting of inward transport, stoichiometry would be expected to be already 2:1 [8], and there is no evidence that secretagogues can reverse it to 3:1 in either of the NBC1 subtypes. Accordingly, Pedrosa et al. did not find any influence of cAMP stimulation on kNBC1 in renal cells in pH_i _recovery experiments [22]; however, it is under debate whether kNBC1 can be a relevant base loader under physiological conditions, a recent study suggesting rather an adaptation of the pNBC1 and kNBC1 expression pattern in renal and submandibular gland epithelia during acid-base disturbances [23]. The differential regulation of SLC4 gene family members in different cell types might be related to possibly specialized roles of the transporters in the respective tissues (e. g. vectorial transport vs. homeostatic functions), but this aspect of NBC function is yet poorly understood.

With respect to the HEK293 cells used in the current study, transfection studies investigating human heart (hhNBC) or renal NBC did not reveal any endogenous expression of the these isoforms by immunoblotting [24,25]. In their functional experiments, one group reported Na^+^- and HCO_3_^-^-dependent pH_i _recovery after an acid load, which could be due to NHE or NBC [24], while another group described no recovery in the additional presence of amiloride, arguing against the presence of endogenous NBC [26]. In support of these findings, the recovery rates we measured in untransfected cells in the presence of CO_2_/HCO_3_^- ^were very low, and, although not statistically comparable since measured in a different experiment, not higher than in its absence. Therefore, the transfection-independent recovery rate is most likely due to endogenous Na^+^/H^+ ^exchangers, of which the NHE1 and NHE3 isoforms have previously been detected in HEK293 cells [15,16]. Since recovery rates were considerably lower in untransfected vs. transfected cells in the presence of CO_2_/HCO_3_^- ^(Figure 4B), no attempt was made to pharmacologically inhibit the presumably relatively low endogenous transporter activities in the subsequent experiments. One limitation of the present study is therefore the lack of information on potential effects of carbachol and forskolin on endogenous base loading mechanisms.

The transfection efficiency was relatively low under all conditions we tried, e. g. serum free/serum containing media, lipophilic agents, or calcium precipitation. Furthermore, a suitable transfection system to serve as a control is not available, since the intestinal cell lines Caco-2 and HT-29 show endogenous NBC activities which are not well molecularly characterized [27-29], T84 cells express kNBC1 rather than pNBC1 (B. Bridges, communication), and our attempts to transfect pNBC1 into Caco-2 or T84 cells did not yield sufficient overexpression to achieve unequivocal results. One possible reason for the overall low transfection efficiency could be that NBC increases intracellular pH_i _above the optimum for cellular metabolism. However, the pH_i _changes we measured were minor, and the acidification of cytosolic pH by lowering medium pH did not improve NBC abundance (data not shown). More likely, transfection efficiency is limited by the large size of the GFP-pNBC1 transcript. To differentiate between transfected and untransfected cells in this setting, the fluorescence signal of the GFP tag was visualized using confocal microscopy. Since the C-terminal phosphorylation site was characterized as a crucial regulatory sequence which explains the regulation of kNBC1 and pNBC1 in renal cells [7,8], it appeared reasonable to tag the pNBC1 N-terminus to avoid interference of the tag with this sequence. Nevertheless, we observed an inhibition of NBC after preincubation with forskolin, which cannot be readily explained by the N-terminal GFP tag. However, we cannot exclude that the GFP tag interferes with the binding site for IRBIT close to the N-terminus (see below), which could in part explain our results with carbachol.

The molecular basis for the differential regulation of pNBC in intestinal vs. HEK293 cells remains unknown. The observed acid-induced NBC activity in HEK293 cells under basal conditions and the strong inhibitory effect of forskolin are difficult to reconcile with mere stoichiometry changes as described by Gross et al. [7], since they would further increase HCO_3_^- ^import and thus accelerate pH_i _recovery. Possibly, the signal transduction machinery of the cell types provides an explanation. One could speculate about a possible role of associated proteins, such as NHERF (NHE regulatory factor) family members, IRBIT (IP_3_R binding protein released with IP_3_), and carbonic anhydrase. NHERF has been shown to be necessary for cAMP-dependent inhibition of renal NBC; however, no binding, NBC phosphorylation or change in NBC surface expression were observed [30,31]. As another regulatory protein, IRBIT was found to bind to pNBC1, but not kNBC1, and pNBC1 transfected into X. oocytes only attains functional activity comparable to the one of transfected kNBC1 when IRBIT is co-transfected [32]. IRBIT is expressed in the kidney [33], while its presence in HEK293 cells and the intestinal tract has not yet been clarified. Furthermore, carbonic anhydrase, of which several isoforms are differentially expressed in the kidney and the gastrointestinal tract [34], could potentially influence NBC transport capacity by HCO_3_^- ^generation, but its functional relevance for NBC regulation is highly controversial at present [35-37].

## Conclusion

In summary, we succeeded in functionally transfecting GFP-pNBC1 into HEK293 cells, thereby providing a non-intestinal cellular model to study its regulation. Na^+^/HCO_3_^-^-cotransporter response to secretagogues in this system was strikingly different from our previous findings in native murine colon. This suggests a relevance of cellular factors, possibly associated proteins, in addition to structural features of this NBC1 variant. The characterization of the involved molecular mechanisms requires further studies.

## Methods

### Construction of tagged full-length pNBC1

Full-length pNBC1 was generated by PCR, using the following sense and antisense primers: 5'-GAATTCAGGATGGAGGATGAAGCTGTCCTG-3' and 5'-AGCGGCCGCCTCAGCATGATGTGTGGCGTTCAAGGAATGT-3', respectively, based on the pancreatic NBC1 cDNA (AF069510). The amplified wild-type NBC1 DNA was fused translationally in-frame to green fluorescent protein (GFP) by cloning into pcDNA3.1/NT-GFP-TOPO vector (Invitrogen, Karlsruhe, Germany).

### Transient expression of epitope-tagged pNBC1 in HEK293 cells

Transfection of HEK293 cells (purchased from the European Collection of Cell Cultures at passage 59) grown to 50% confluence on 35 mm tissue culture plates was performed either with the calcium phosphate method following Jordan et al. [38] using 6 μg expression construct DNA per dish, or with Lipofectamine 2000 (Invitrogen, Karlsruhe, Germany) according to the manufacturer's instructions using 4 μg expression construct DNA and 12 μl Lipofectamine per dish for the pNBC1 construct (2 μg and 8 μl for the vector alone).

### Polymerase chain reaction (PCR) for pNBC1 and kNBC1

The primers used for amplifying pNBC1 and kNBC1 were deduced from published sequence information [9,26], and from PCR protocols with murine samples [18] (5'-ATGTGTGTGATGAAGAAGAAGTAGAAG-3' and 5'-CACTGAAAATGTGGAAGGGAAG-3', respectively, as well as the common antisense primer 5'-GACCGAAGGTTGGATTTCTTG-3'). The following conditions were used for PCR: denature 94°C, 30 s; anneal 58°C, 30 s; extend 72°C, 180 s; for 30 cycles.

### Determination of NBC activity by confocal microscopy

Transiently transfected HEK293 cells grown on a glass coverslip were transferred to a custom-made perfusion chamber and mounted on the heated stage of a fixed-stage upright confocal microscope (Leica DM LFSA, Leica TCS SP2 AOBS, Leica Microsystems, Bensheim, Germany) 48–72 h post transfection. After documentation of the GFP fluorescence, cells were loaded with 5 μM BCECF for 30 minutes in buffer A (120 NaCl, 14 HEPES, 7 TRIS, 3 KH_2_PO_4_, 2 K_2_HPO_4_, 1.2 MgSO_4_, 1.2 Ca^2+^-gluconate, 20 glucose, pH 7.4, gassed with 100% O_2_), and subsequently perfused with 95% O_2_/5% CO_2_-gassed buffers (buffer B: 20 mM NaCl of buffer A were replaced by NaHCO_3_; buffer C: 40 mM NaCl of buffer B were replaced by NH_4_Cl; buffer D: NaCl of buffer A was replaced by tetramethyl-ammonium chloride) following the respective protocol. The fluorescence ratio was recorded during alternating excitation with a 442 nm diode-pumped solid-state (DPSS) laser system and the 488 nm line of the built-in argon laser. The system's Acousto-Optical Beam Splitter (AOBS) was set to an emission range of 525 ± 10 nm. Subsequent 2-point calibration (pH 6.2 and 7.0) was carried out using the high-K^+^-nigericin method as described previously [6] to enable calculation of pH_i _from the ratio values for each individual ROI. These calibration points were chosen since the primary aim was to measure the steep initial pH_i _recovery rate from the reference pH_i _(Fig. 3B), and the slope of the calibration curve was found to be less steep in this pH range (Fig. 2C). GFP fluorescence was negligible compared to the BCECF signal (<1%).

### Materials

2'-7'-bis-carboxyethyl-5,6-carboxyfluorescein acetoxymethyl ester (BCECF/AM) was purchased from Invitrogen (Karlsruhe, Germany), and cell culture media (DMEM) as well as fetal calf serum from Fluka (Seelze, Germany). Forskolin, carbachol and all other chemicals were purchased from Sigma-Aldrich (Seelze, Germany), and were either of cell culture grade or the highest grade available.

### Statistics

Results of the pH_i _recovery rates are given as means± SE (standard error), except for the box plot shown in figure 3 displaying the median and quartiles. Since transfected and untransfected cells were measured on the same plate in each individual experiment, statistical testing between the two was carried out using student's t-test in its paired form; otherwise, its unpaired form was used. For the calculation of standard errors and for statistics, n = 1 was defined as the mean of all ROIs (transfected or untransfected) from one experiment.

## Authors' contributions

OB participated in the design and the coordination of the study, performed the statistical analysis, and drafted the manuscript. KF carried out the transfections, the initial PCR assays, and the fluorometric experiments. HY performed the final PCR assays and participated in the fluorometric experiments. BR participated in optimizing the PCR and the transfection. HCL and MS cloned the expression construct. MPM participated in the coordination of the study. US participated in the design of the study and revised the manuscript. All authors read and approved the final manuscript.
